# Quality improvement project: to improve adherence to DVLA (driving and vehicle liscensing agency) guidance in the tyrone & fermanagh hospital acute inpatients ward

**DOI:** 10.1192/bjo.2021.591

**Published:** 2021-06-18

**Authors:** Vivian Sing, Chad Ballantine, Peter Hackett

**Affiliations:** 1 Ulster Hospital; 2 Tyrone and Fermanagh Hospital

## Abstract

**Aims:**

To reach 80% adherence to DVANI (Driving and Vehicle Agency Northern Ireland) guidance in acute inpatients ward, T&F Hospital

**Background:**

This is a scale-up of a previous successful QI project on driving and Attention Deficit Hyperactivity Disorder in Belfast Trust. According DVLA's guidance for medical practitioners on the current medical standards of fitness to drive, patients with certain mental health diagnosis are required to inform DVLA of their diagnoses and refrain from driving. Different factors are considered in order to determine patients’ fitness to drive. According to DVLA and GMC, it is medical professionals’ responsibility to advise patients to inform DVLA/DVANI of their mental health diagnosis. It is the patient's legal duty to notify DVLA/DVANI of their diagnosis. Patients can be fined up to £1000 if they failed to inform DVANI of their medical condition.

**Method:**

Outcome: Completeness of driving advice given to consecutive patients discharged from T&F hospital from April 2019 to early August 2019 in %

Process: Document clearly in electronic and written notes on following - (1) has driving status been asked (2) has patient been advised to inform DVA if required (3) has patient been advised likely how long he/she is to refrain from driving for

Balancing: increased the time of reviews, increased numbers of consultant reports requested from DVA

**Result:**

4 cycles have been completed. Cycle 1 – baseline and review guidance; Cycle 2 – medical staff education and developed driving advice pathway and patient leaflet; Cycle 3 – admin staff was involved for putting driving advice pathway in admission pack; Cycle 4 – medical staff was educated again regarding importance of documenting electronically. Clear changes were seen after cycle 3 showing an increase of mean of 25% completeness of driving advice to over 90%.

**Conclusion:**

It is the legal duty of patients to notify DVANI of mental health diagnosis, however it is the responsibility of medical professionals to advise patients to do so. This QI project has shown improvement in the completeness of driving advice given. Further cycles are to be completed to obtain patient feedback.

